# Severe Illness from Methyl Bromide Exposure at a Condominium Resort — U.S. Virgin Islands, March 2015

**DOI:** 10.15585/mmwr.mm6428a4

**Published:** 2015-07-24

**Authors:** Prathit A. Kulkarni, Mary Anne Duncan, Michelle T. Watters, Leah T. Graziano, Elena Vaouli, Larry F. Cseh, John F. Risher, Maureen F. Orr, Tai C. Hunte-Ceasar, Esther M. Ellis

**Affiliations:** 1 Epidemic Intelligence Service, CDC; 2 Division of Toxicology and Human Health Sciences, Agency for Toxic Substances and Disease Registry; 3 Division of Community Health Investigations, Agency for Toxic Substances and Disease Registry; 4 U.S. Virgin Islands Department of Health

On March 22, 2015, the Agency for Toxic Substances and Disease Registry (ATSDR) was notified by the U.S. Environmental Protection Agency (EPA) of four cases of suspected acute methyl bromide toxicity among family members vacationing at a condominium resort in the U.S. Virgin Islands. Methyl bromide is a pesticide that has been banned in the United States for use in homes and other residential settings. An investigation conducted by the U.S. Virgin Islands Department of Health (VIDOH), the U.S. Virgin Islands Department of Planning and Natural Resources (DPNR), and EPA confirmed that methyl bromide had been used as a fumigant on March 18 in the building where the family had been residing, 2 days before they were transported to the hospital; three family members had life-threatening illness. On March 25, 2015, a stop-use order for methyl bromide was issued by DPNR to the pest control company that had performed the fumigation. Subsequent investigation revealed that previous fumigation with methyl bromide had occurred on October 20, 2014, at the same condominium resort. In addition to the four ill family members, 37 persons who might have been exposed to methyl bromide as a result of the October 2014 or March 2015 fumigations were identified by VIDOH and ATSDR. Standardized health questionnaires were administered to 16 of the 20 persons for whom contact information was available; six of 16 had symptoms consistent with methyl bromide exposure, including headache and fatigue. Pest control companies should be aware that use of methyl bromide is banned in homes and other residential settings, and clinicians should be aware of the toxicologic syndrome that exposure to methyl bromide can cause.

## Illness Cluster Identification and Background

On March 20, 2015, a family of four vacationing in the U.S. Virgin Islands was transported to a hospital with a 24-hour history of progressive neurologic symptoms, including generalized weakness, severe myoclonus, fasciculations, altered sensorium, and word-finding difficulty. Three of the four patients had vomiting and diarrhea; three required endotracheal intubation and mechanical ventilation.

Initial clinical suspicion included organophosphate toxicity and ciguatera fish poisoning; however, other persons who had consumed the same food as the patients had not become ill. Because chemical toxicity was considered as a possible etiology for the family’s illness, management at the condominium resort where the family had been residing was contacted. A preliminary investigation revealed that an unoccupied housing unit below the one in which the family had been staying, but in the same building, had been fumigated with the pesticide methyl bromide on March 18, 2 days before the family sought medical care. The patients’ neurologic syndrome was consistent with acute methyl bromide toxicity.

The family consisted of two teens and two adults, with an age range from 14 to 49 years. All four patients were treated with benzodiazepines, phenobarbital, and propofol for sedation and symptom control. The two teens were treated with the neuromuscular blocking agent rocuronium because of symptom severity, and the two adults received pralidoxime because of the initial suspicion of possible organophosphate toxicity. All four patients underwent two hemodialysis procedures when methyl bromide toxicity became the leading diagnostic consideration. Serum bromide levels of the patients obtained on March 20, the day the family was taken to the hospital, ranged from <10 mg/dL* to 13.6 mg/dL (of note, three of four specimens for bromide level determination were obtained after initiation of dialysis).

Within 3 days, all four patients were transferred to hospitals in the continental United States for further care. As of June 30, all four patients had been discharged from acute-care hospitals. Three of the four patients were receiving inpatient physical rehabilitation for significant neurologic dysfunction.

Methyl bromide was first reported to be effective as a pesticide in 1932, and was first registered for use as such in the United States in 1961 (1). Currently, its primary use is in agricultural settings for soil fumigation and in greenhouses, warehouses, and ships (2). Beginning in 1993, on the basis of the U.S. Clean Air Act and the Montreal Protocol on Substances that Deplete the Ozone Layer (1992), use of methyl bromide in the United States was set at 1991 baseline levels (3). During 1999–2005, a gradual and planned reduction in the use of methyl bromide because of its ozone-depleting properties was implemented. Certain exemptions for the use of methyl bromide are permitted, including a critical-use exemption and a quarantine and preshipment exemption; neither of these exemptions, however, allows for the use of methyl bromide in residential settings.

Mild symptoms from acute inhalational exposure to methyl bromide include headache, malaise, generalized weakness, and nausea; more severe manifestations of acute inhalational exposure include tremors, myoclonus, altered mental status, seizures, respiratory symptoms, and renal failure (4). Symptoms from acute inhalational exposure generally appear within 48 hours (4). During 1899–1981, a total of 115 fatal and 843 nonfatal cases of methyl bromide toxicity were reported worldwide (5). In general, with inhalational exposure, appearance of clinical symptoms depends on the air concentration of methyl bromide to which a person is exposed and the duration of exposure at that particular level. The EPA Office of Pesticide Programs considers acute inhalational exposure to methyl bromide for 1 day to be of concern at the following levels: ≥0.33 parts per million (ppm) for a 24-hour time-weighted average in nonoccupational settings and ≥1 ppm for an 8-hour time-weighted average in occupational settings (6).

Methyl bromide exposure can be evaluated by measuring the bromide ion level in serum. However, detected levels of bromide ion do not always correlate with the presence or severity of clinical symptoms. A bromide level of <1.5 mg/dL is considered normal (7).† Levels as high as 8 mg/dL have been detected in the absence of overt clinical signs or symptoms (7). Conversely, one report documented chronic toxicity at serum levels as low as 4.4 mg/dL (8).

## Investigation and Public Health Response

After methyl bromide had been identified as the likely cause of the patients’ symptoms, both the fumigated lower unit and the upper unit in which the family had been residing were immediately sealed off. No other housing units were in the building. An assessment by DPNR and EPA confirmed that only methyl bromide had been used for fumigation. Sampling results from EPA taken on March 24 revealed the air concentration of methyl bromide to be 1.12 ppm in the upper housing unit; on March 27, the concentration was 0.59 ppm in the lower housing unit. Although these levels are elevated, it is not possible to know the specific air concentration of methyl bromide the family had been exposed to during March 18–20, because sampling was not done at the time fumigation was performed. An investigation by DPNR and EPA revealed that methyl bromide had also been used at the same condominium complex by the same pest control company on October 20, 2014, in four additional housing units. Air measurements were unavailable for the October 2014 fumigation. As a result of the investigation, on March 25, 2015, DPNR issued an immediate stop-use order to the pest control company that had performed the fumigations; the stop-use order prevented any further use of methyl bromide by the company in any setting in the U.S. Virgin Islands. EPA, in coordination with DPNR and VIDOH, subsequently performed monitoring and ventilation of the building fumigated in March 2015.

VIDOH and ATSDR, working with EPA and condominium management, sought to identify persons in addition to the affected family who had potentially been exposed to methyl bromide at the condominium resort to more fully characterize health effects of the fumigations. The identification process included any person who had been inside a fumigated building during the 2 weeks following the date of fumigation (or until the building was sealed off in the case of the March 2015 fumigation). This 2-week time frame was based on two primary considerations: 1) preliminary results of environmental sampling over time; and 2) expected decreases in the air concentration of methyl bromide in the affected housing units and in the associated risk of adverse health effects (6,9). Potentially exposed persons included pest control company personnel, emergency responders, condominium staff members, and resort residents, vacationers, and visitors. A standardized questionnaire was used to interview exposed persons about possible exposure-related health effects.

## Other Potential Exposures

In addition to the family of four, 37 persons were identified who had potentially been exposed to methyl bromide; 11 were potentially exposed after the March 2015 fumigation, 20 after the October 2014 fumigation, and one during both periods. Whether the remaining five persons were exposed during one or both periods is unknown. Contact information was unavailable for 17 of the 37 persons; among the 20 for whom contact information was available, 16 were surveyed. Of the 16, eight had been exposed after the March 2015 fumigation, seven after the October 2014 fumigation, and one during both periods.

No persons exposed only after the October 2014 fumigation reported any adverse health effects. Among the eight persons exposed after the March 2015 fumigation and the one person exposed during both periods, six had postexposure symptoms. Among these nine persons, maximum exposure time was approximately 75 minutes after the March 2015 fumigation. All six persons who had symptoms reported headache, and four reported fatigue. One person had shortness of breath, and another had a cough. All symptoms resolved within 3 weeks of exposure except for certain symptoms experienced by two persons, one who had a persistent mild headache and another who had a mild cough that had been present before the exposure occurred. Four of the six persons who had postexposure symptoms were emergency responders. In addition, four exposed persons had serum levels of bromide measured on March 23, 2015, 3 days after their exposure; bromide ion values ranged from <10 mg/dL to 15.3 mg/dL. Three of these four persons had symptoms.

### Discussion

Methyl bromide toxicity has become less common after the use of methyl bromide began to be curtailed during the 1990s; the most recent report of methyl bromide toxicity was published in 2011 (8). However, systematic surveillance for methyl bromide toxicity does not occur on a national level, so it is difficult to know if cases have not occurred or have not been recognized or reported.

This investigation serves as a reminder to clinicians to consider the possibility of acute chemical toxicity in the relevant clinical and epidemiologic situation (e.g., an isolated family of four with rapid progression of a distinct clinical syndrome). Prompt identification of such an exposure, as occurred in this situation, can prevent further exposure and subsequent illness. Interpretation of bromide levels should be undertaken cautiously and with the assistance of personnel trained in toxicology or occupational health because of limitations of certain types of bromide testing and the lack of direct correlation between bromide levels and the presence and severity of clinical symptoms.

This investigation also highlights the public health consequences of methyl bromide use in a residential setting. Certain unsuspecting persons were exposed to this highly toxic chemical because of its nonpermitted use. Of note, most persons who experienced postexposure symptoms were emergency responders to the scene. At the time they responded, a chemical exposure was not known to be the definitive cause of the family’s illness. However, if a toxic chemical release is ever identified, subsequent prompt notification of potentially exposed emergency responders is recommended.

Use of methyl bromide is restricted to specified settings as required by U.S. law and international regulations. Pest control companies should be aware of all rules regarding products they use and should ensure that all staff members receive proper education and training for application of these products in a safe manner, including use of personal protective equipment and appropriate signage. In addition, pest control companies and others involved in environmental pest control should consider the use of integrated pest management, which emphasizes prevention measures and understanding of pest life cycles and the interaction of pests with their environment (10). Broadcast spraying with nonspecific pesticides is used judiciously, if at all, in integrated pest management.

**Summary**

What is already known on this topic?

Methyl bromide is a highly toxic pesticide which is banned in the United States for use in homes and other residential settings. Acute toxicity from methyl bromide can result in serious neurologic disease or even death.

What is added by this report?

During March 2015, a family of four vacationing in the U.S. Virgin Islands developed acute methyl bromide toxicity as a result of improper use of methyl bromide by a pest control company at a residential location. An investigation revealed that the same company had used methyl bromide at the same location 5 months earlier. Thirty-seven additional persons were identified as potentially having been exposed to methyl bromide as a result of these fumigations. Six persons, four of whom were emergency responders, developed postexposure symptoms, including headache and fatigue.

What are the implications for public health practice?

Clinicians should be alert to the possibility of chemical toxicity in the appropriate clinical and epidemiologic setting. Pest control companies should be aware of all laws and regulations surrounding the use of highly toxic chemicals. Correct safety protocols should be followed, and adequate training for applicators should be provided.

## Footnotes

* Values of <10 mg/dL are beyond the validated reporting limit of the particular assay that was used to determine serum bromide levels.

† A normal serum bromide level might not be zero because of routine exposure to a limited amount of bromide from diet, medications, or other sources.
